# Supplementary material for: A PPARG Splice Variant in Granulosa Cells Is Associated with Polycystic Ovary Syndrome
Source: J Clin Med. 2022 Dec 8;11(24):7285. doi: 10.3390/jcm11247285 (PMC9786670; doi:10.3390/jcm11247285)
Supplement: Supplementary file 1 [file jcm-11-07285-s001.zip › jcm-1986925-supplementary.pdf]

Supplementary

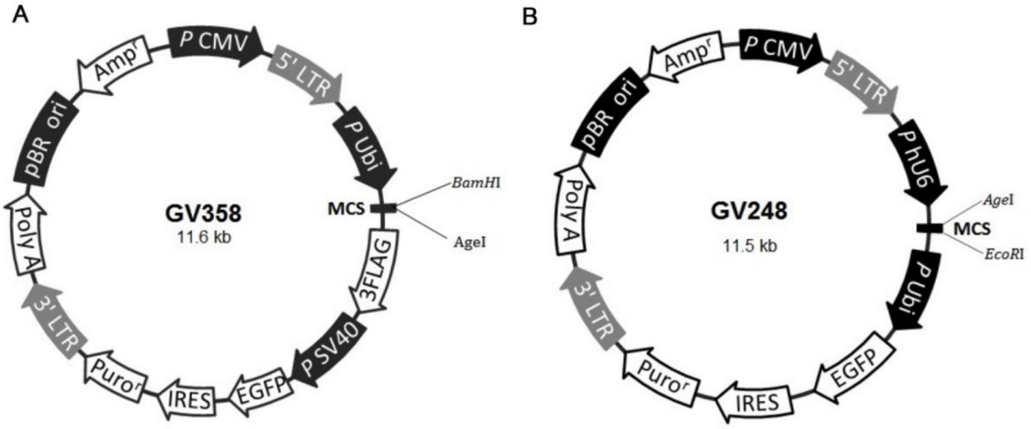

Figure S1. Vectors for lentiviral construction.

(A) rPPARG

Vector information：

General：11615bp

HIV-1_5_LTR, trunkHIV-1_3_LTR：835-1015

psi：1067-1204

RRE：1680-1913

ORF frame 1：1558-2445

Ubiquitin Promoter：2617-3833

3FALG：3880-3957

SV40 promoter：3964-4341

EGFP：4350-5069

IRES：5092-5676

Puromycin：5683-6282

WPRE：6309-6896

HIV-1_5_LTR, trunkHIV-1_3_LTR：7420-7600

pBR322_origin：10467-9848

Ampicillin：11482-10622

AmpR_promoter：11552-11524

CAG_enhancer：318-605

CMV_immearly_promoter：239-810

Primer locations and sequences：

Ubi-F（3756-3778）：GGGTCAATATGTAATTTTCAGTG

FLAG-R-1（3963-3943）：GCTAGCTCATTTGTCGTCATC

The insertion sequence is shown in red, the vector sequence is shown in black, and the restriction site is underlined.

CGTTTTGGCTTTTTGTTAGACGAAGCTTGGGCTGCAGGTCGACTCTAGAGGATCCCCGGGTACCGGTCGCCACCATGGGT
GAAACTCTGGGAGATTCTCCTATTGACCCAGAAAGCGATTCCTTCACTGATACACTGTCTGCAAACATATCACAAGAAATG
ACCATGGTTGACACAGAGATGCCATTCTGGCCCACCAACTTTGGGATCAGCTCCGTGGATCTCTCCGTAATGGAAGACCAC
TCCCACTCCTTTGATATCAAGCCCTTCACTACTGTTGACTTCTCCAGCATTTCTACTCCACATTACGAAGACATTCCATTCAC
AAGAACAGATCCAGTGGTTGCAGATTACAAGTATGACCTGAAACTTCAAGAGTACCAAAGTGCAATCAAAGTGGAGCCT
GCATCTCCACCTTATTATTCTGAGAAGACTCAGCTCTACAATAAGCCTCATGAAGAGCCTTCCAACTCCCTCATGGCAATT
GAATGTCGTGTCTGTGGAGATAAAGCTTCTGGATTTCACTATGGAGTTCATGCTTGTGAAGGATGCAAGGGTTTCTTCCGGA
GAACAATCAGATTGAAGCTTATCTATGACAGATGTGATCTTAACTGTCGGATCCACAAAAAAAGTAGAAATAAATGTCAG
TACTGTCGGTTTCAGAAATGCCTTGCAGTGGGGATGTCTCATAATGCCATCAGGTTTGGGCGGATGCCACAGGCCGAGAA
GGAGAAGCTGTTGGCGGAGATCTCCAGTGATATCGACCAGCTGAATCCAGAGTCCGCTGACCTCCGGGCCCTGGCAAAAC
ATTTGTATGACTCATACATAAAGTCCTTCCCGCTGACCAAAGCAAAGGCGAGGGCGATCTTGACAGGAAAGACAACAGA
CAAATCACCATTCGTTATCTATGACATGAATTCCTTAATGATGGGAGAAGATAAAATCAAGTTCAAACACATCACCCCCCT
GCAGGAGCAGAGCAAAGAGGTGGCCATCCGCATCTTTCAGGGCTGCCAGTTTCGCTCCGTGGAGGCTGTGCAGGAGATCA
CAGAGTATGCCAAAAGCATTCCTGGTTTTGTAAATCTTGACTTGAACGACCAAGTAACTCTCCTCAAATATGGAGTCCACG
AGATCATTTACACAATGCTGGCCTCCTTGATGAATAAAGATGGGGTTCTCATATCCGAGGGCCAAGGCTTCATGACAAGG
GAGTTTCTAAAGAGCCTGCGAAAGCCTTTTGGTGACTTTATGGAGCCCAAGTTTGAGTTTGCTGTGAAGTTCAATGCACTG
GAATTAGATGACAGCGACTTGGCAATATTTATTGCTGTCATTATTCTCAGTGGAGACCGCCCAGGTTTGCTGAATGTGAAG
CCCATTGAAGACATTCAAGACAACCTGCTACAAGCCCTGGAGCTCCAGCTGAAGCTGAACCACCCTGAGTCCTCACAGCT
GTTTGCCAAGCTGCTCCAGAAAATGACAGACCTCAGACAGATTGTCACGGAACACGTGCAGCTACTGCAGGTGATCAAGA
AGACGGAGACAGACATGAGTCTTCACCCGCTCCTGCAGGAGATCTACAAGGACTTGTACGGTATGGACTACAAGGATGAC
GATGACAAGGATACAAAGACGACGA

(B) shPPARG

GV248
Vector information：
General：11468bp
HIV-1_5_LTR, truncHIV-1_3_LTR：835-1015
psi：1067-1204
RRE：1680-1913
ORF frame 1：1558-2445
hU6Promoter：2602-2869
me-shid-2: 2938-2961
Ubiquitin Promoter：2957-4173
EGFP：4216-4935
IRES：4945-5529
Puromycin：5536-6135
WPRE：6149-6736
HIV-1_5_LTR, truncHIV-1_3_LTR：7247-7427
pBR322-origin：10294-9675
AmpR：11309-10449
AmpR-promoter：11379-11351
CAG_enhance：318-605

CMV_immearly_promoter：239-810

Primer locations and sequences：

H1-F(2479-2502)：GGAAAGAATAGTAGACATAATAGC

Ubi-R(3136-3115)：ATGTCCTTCTGCTGATACTGGG

**Target Seq**	**GC%**
GACCAAGTAACTCTCCTCAAA	42.86%

**NO.**	**5′**	**STEM**	**Loop**	**STEM**	**3′**
pparg1-RNAi(63592-1)-a	Ccgg	GACCAAGTAACTCTCCTCAAA	CTCGAG	TTTGAGGAGAGTTACTTGGTC	TTTTTg
pparg1-RNAi(63592-1)-b	aattcaaaaa	GACCAAGTAACTCTCCTCAAA	CTCGAG	TTTGAGGAGAGTTACTTGGTC	

F_C02.ab1NNNNNNN

AACTAAAGATTACAAAAACAAATTACAAAAATTCAAAATTTTCGGGTTTATTACAGGGACAGCAGAGATCCAGTTTGGTT
AATTAATCGAGCGGCCGCCCCCTTCACCGAGGGCCTATTTCCCATGATTCCTTCATATTTGCATATACGATACAAGGCTGTT
AGAGAGATAATTGGAATTAATTTGACTGTAAACACAAAGATATTAGTACAAAATACGTGACGTAGAAAGTAATAATTTCT
TGGGTAGTTTGCAGTTTTAAAATTATGTTTTAAAATGGACTATCATATGCTTACCGTAACTTGAAAGTATTTCGATTTCTTGG
CTTTATATATCTTGTGGAAAGGACGAAACACCGGGACCAAGTAACTCTCCTCAAACTCGAGTTTGAGGAGAGTTACTTGGT
CTTTTTGAATTCTCGACCTCGAGACAAATGGCAGTATTCATCCACGGATCCTAACCCGTGTCGGCTCCAGATCTGGCCTCCG
CGCCGGGTTTTGGCGCCTCCCGCGGGCGCCCCCCTCCTCACGGCGAGCGCTGCCACGTCAGACGAAGGGCGCAGCGAGCG
TCCTGATCCTTCCGCCCGGACGCTCAGGACAGCGGCCCGCTGCTCATAAGACTCGGCCTTAGAACCCCAGTATCAGCAGA
AGGACATTTTAGGACGGGACTTGGGTGACTCTAGGGCACTGGTTTTCTTTCCAGAGAGCGGAACAGGCGAGGAAAAGTAG
TCCCTTCTCGGCGATTCTGCGGAGGGATCTCCGTGGGGCGGTGAACGCCGATGATTATATAAGGACGCGCCGGGTGTGGC
ACAGCTAGTTCCGTCGCAGCCGGGATTTGGGTCGCGGTTCTTGTTTGTGGATCGCTGTGATCGTCACTTGGTGAGTAGCGGG
CTGCTGGGCTGGCCGGGGCTTTCGTGGCCGCCGGGCCGCTCGGTGGGACGGAAGCGTGTGGAGAGACCGCCAAGGGCTGT
AGTCTGGGTCCGCGAGCAAGGTTGCCCTGAACTGGGGGTTGGGGGGANCGCAGCAAATGNNGCTGTTCCCGAGTCTTGAT
GNNGACGCTTGTGAGCGGGCTGTGAGGTCGTGAAACAGGTGGGGGGCATGGTGGGGCGGCAGACCNNNCNTGGAGGCC
TTCGCTAA

PSC63592-1 ccggGACCAAGTAACTCTCCTCAAActcgagTTTGAGGAGAGTTACTTGGTCtttttg

（C）shPPARG+rPPARG SV

The coding sequence of the rPPARG SV was cloned into the LV6 vector with psi-RRE-EF1a-puromycin-WPRE and then co-transfected with shPPARG lentiviruses into KGN cells to produce lentiviruses overexpressing the PPARG SV and weakly expressing normal PPARG.

The upstream and downstream primers of the target gene are respectively added with homologous sequences on both sides of NotI and BamHI on LV6 vector for subcloning of the vector. The primer sequences are as follows:

B4537-1	AGGGTTCCAAGCTTAAGCGGCCGCATTTGA
B4537-2	TGAGAAAATGGCCTTGTTGTATATTTGTGGTTTAGTGTTGGCTTCTTTCAAATGCGGCCGCTTAA
B4537-3	AATATACAACAAGGCCATTTTCTCAAACGAGAGTCAGCCTTTAACGAAATGACCATGGTTGACAC
B4537-4	CCACGGAGCTGATCCCAAAGTTGGTGGGCCAGAATGGCATCTCTGTGTCAACCATGGTCATTTCG
B4537-5	TGGGATCAGCTCCGTGGATCTCTCCGTAATGGAAGACCACTCCCACTCCTTTGATATCAAGCCCT
B4537-6	TTCGTAATGTGGAGTAGAAATGCTGGAGAAGTCAACAGTAGTGAAGGGCTTGATATCAAAGGAGT
B4537-7	GCATTTCTACTCCACATTACGAAGACATTCCATTCACAAGAACAGATCCAGTGGTTGCAGATTAC
B4537-8	ACTTTGATTGCACTTTGGTACTCTTGAAGTTTCAGGTCATACTTGTAATCTGCAACCACTGGATC
B4537-9	GAGTACCAAAGTGCAATCAAAGTGGAGCCTGCATCTCCACCTTATTATTCTGAGAAGACTCAGCT
B4537-10	CATGAGGGAGTTGGAAGGCTCTTCATGAGGCTTATTGTAGAGCTGAGTCTTCTCAGAATAATAAG
B4537-11	GCCTTCCAACTCCCTCATGGCAATTGAATGTCGTGTCTGTGGAGATAAAGCTTCTGGATTTCACT
B4537-12	CCGGAAGAAACCCTTGCATCCTTCACAAGCATGAACTCCATAGTGAAATCCAGAAGCTTTATCTC
B4537-13	ATGCAAGGGTTTCTTCCGGAGAACAATCAGATTGAAGCTTATCTATGACAGATGTGATCTTAACT
B4537-14	GTACTGACATTTATTTCTACTTTTTTTGTGGATCCGACAGTTAAGATCACATCTGTCATAGATAA
B4537-15	ACAAAAAAAGTAGAAATAAATGTCAGTACTGTCGGTTTCAGAAATGCCTTGCAGTGGGGATGTCT
B4537-16	TCTCCTTCTCGGCCTGTGGCATCCGCCCAAACCTGATGGCATTATGAGACATCCCCACTGCAAGG
B4537-17	CACAGGCCGAGAAGGAGAAGCTGTTGGCGGAGATCTCCAGTGATATCGACCAGCTGAATCCAGAG
B4537-18	TGTATGAGTCATACAAATGTTTTGCCAGGGCCCGGAGGTCAGCGGACTCTGGATTCAGCTGGTCG
B4537-19	GCAAAACATTTGTATGACTCATACATAAAGTCCTTCCCGCTGACCAAAGCAAAGGCGAGGGCGAT
B4537-20	CATTCAGCAAACCTGGGCGGTTGATTTGTCTGTTGTCTTTCCTGTCAAGATCGCCCTCGCCTTTG
B4537-21	GCCCAGGTTTGCTGAATGTGAAGCCCATTGAAGACATTCAAGACAACCTGCTACAAGCCCTGGAG
B4537-22	TTGGCAAACAGCTGTGAGGACTCAGGGTGGTTCAGCTTCAGCTGGAGCTCCAGGGCTTGTAGCAG
B4537-23	CCTCACAGCTGTTTGCCAAGCTGCTCCAGAAAATGACAGACCTCAGACAGATTGTCACGGAACAC
B4537-24	CTCATGTCTGTCTCCGTCTTCTTGATCACCTGCAGTAGCTGCACGTGTTCCGTGACAATCTGTCT
B4537-25	GAAGACGGAGACAGACATGAGTCTTCACCCGCTCCTGCAGGAGATCTACAAGGACTTGTACTAGC
B4537-26	ACTGGAAGAAGGGAAATGTTGGCAGTGGCTCAGGACTCTCTGCTAGTACAAGTCCTTGTAGATCT
B4537-27	CCAACATTTCCCTTCTTCCAGTTGCACTATTCTGAGGGAAAATCTGACACCTAAGAAATTTACTG
B4537-28	CATATTCTAAAACCTTTTCTTTTTAAAATGCTTTTTCACAGTAAATTTCTTAGGTGTCAGATTTT
B4537-29	GCATTTTAAAAAGAAAAGGTTTTAGAATATGATCTATTTTATGCATATTGTTTATAAAGACACAT
B4537-30	TAATATGGTAATTTTTAATATTAAAAGTAAATTGTAAATGTGTCTTTATAAACAATATGCATAAAA
B4537-31	TTACAATTTACTTTTAATATTAAAAATTACCATATTATGAAATTGCTGATAGTAAAAAAAAAAAAA
B4537-32	ATCAGTAGAGAGTGTCGGATCCTTTTTTTTTTTTTTTTTACTATCAGCAATTTCA

Then the target gene PPARG SV was cloned into vector LV6.

The insertion sequence is shown as follows.

ATTTGAAAGAAGCCAACACTAAACCACAAATATACAACAAGGCCATTTTCTCAAACGAGAGTCAGCCTTTAACGAAATG
ACCATGGTTGACACAGAGATGCCATTCTGGCCCACCAACTTTGGGATCAGCTCCGTGGATCTCTCCGTAATGGAAGACCAC
TCCCACTCCTTTGATATCAAGCCCTTCACTACTGTTGACTTCTCCAGCATTTCTACTCCACATTACGAAGACATTCCATTCAC
AAGAACAGATCCAGTGGTTGCAGATTACAAGTATGACCTGAAACTTCAAGAGTACCAAAGTGCAATCAAAGTGGAGCCT
GCATCTCCACCTTATTATTCTGAGAAGACTCAGCTCTACAATAAGCCTCATGAAGAGCCTTCCAACTCCCTCATGGCAATT

GAATGTCGTGTCTGTGGAGATAAAGCTTCTGGATTTCACTATGGAGTTCATGCTTGTGAAGGATGCAAGGGTTTCTTCCGGA
GAACAATCAGATTGAAGCTTATCTATGACAGATGTGATCTTAACTGTCGGATCCACAAAAAAAGTAGAAATAAATGTCAG
TACTGTCGGTTTCAGAAATGCCTTGCAGTGGGGATGTCTCATAATGCCATCAGGTTTGGGCGGATGCCACAGGCCGAGAA
GGAGAAGCTGTTGGCGGAGATCTCCAGTGATATCGACCAGCTGAATCCAGAGTCCGCTGACCTCCGGGCCCTGGCAAAAC
ATTTGTATGACTCATACATAAAGTCCTTCCCGCTGACCAAAGCAAAGGCGAGGGCGATCTTGACAGGAAAGACAACAGA
CAAATCAACCGCCCAGGTTTGCTGAATGTGAAGCCCATTGAAGACATTCAAGACAACCTGCTACAAGCCCTGGAGCTCCA
GCTGAAGCTGAACCACCCTGAGTCCTCACAGCTGTTTGCCAAGCTGCTCCAGAAAATGACAGACCTCAGACAGATTGTCA
CGGAACACGTGCAGCTACTGCAGGTGATCAAGAAGACGGAGACAGACATGAGTCTTCACCCGCTCCTGCAGGAGATCTA
CAAGGACTTGTACTAGCAGAGAGTCCTGAGCCACTGCCAACATTTCCCTTCTTCCAGTTGCACTATTCTGAGGGAAAATCT
GACACCTAAGAAATTTACTGTGAAAAAGCATTTTAAAAAGAAAAGGTTTTAGAATATGATCTATTTTATGCATATTGTTTA
TAAAGACACATTTACAATTTACTTTTAATATTAAAAATTACCATATTATGAAATTGCTGATAGTAAAAAAAAAAAAAAAA
AA

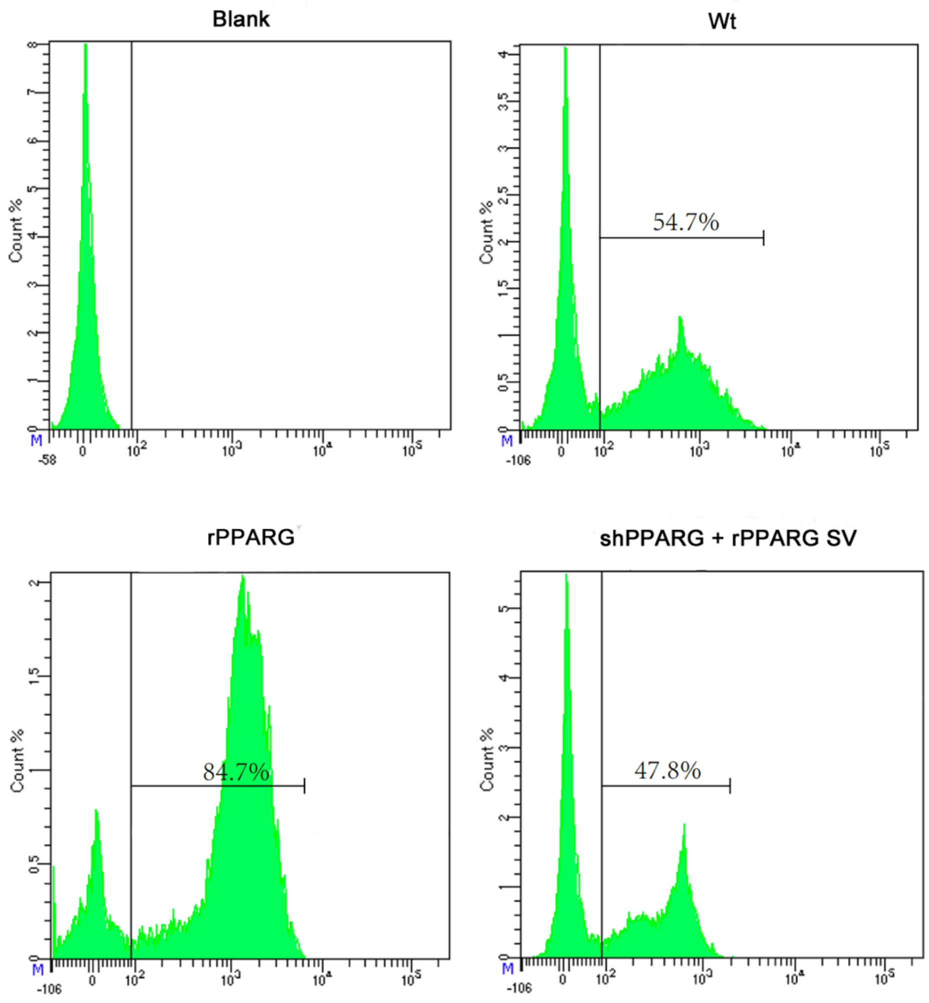

Figure S2. The apoptosis ratio of KGN cells treated with different lentivirus in each group was detected by flow cytometry using TUNEL staining. The figure shows the apoptosis rate decreased following the transfection of PPARG SV.

Table S1. The primers for PCR.

Category	target sequence	specific primer	sequence（5′ to 3′）
Primers for SMARTer RACE	full length of PPARG	SMARTer RACE 3′RT primer-1	AAG GGA AAT GTT GGC AGT GG
		SMARTer RACE 3′RT primer-2	GAT CTC CTG CTG CAG GAG CGG GTG
		SMARTer RACE 3′RT primer-3	CTG TCT CCG TCT TCT TGA TCA CCT GC
		SMARTer RACE3′RT primer-4	GTG CAA CTG GAA GAA CTC TCT GCT AG
Primers for qRT PCR	PPARG exon5	PPARG-ex5-F-1	ACA TAA AGT CCT TCC CGC TG
		PPARG-ex5-R-1	AGC AGG TTG TCT TGA ATG TCT
	PPARG exon5 del	PPARG ex5 Del qPCR Primer1 F	CGA GAA GGA GAA GCT GTT GG
		PPARG ex5 Del qPCR Primer1 R	GCG GTT GAT TTG TCT GTT GT
	ACTB	ACTB-F	AGC GAG CAT CCC CCA AAG TT
		ACTB-R	GGG CAC GAA GGC TCA TCA TT
Primers for PCR	PPARG exon1	PPARG-ex1-F-1	ACA AGG CCA TTT TCT CAA AC
		PPARG-ex1-R-1	TAA GGT GGA GAT GCA GGC TC
		PPARG-ex1-F-2	ACC ACA AAT ATA CAA CAA GGC CA
		PPARG-ex1-R-2	CAG GCT CCA CTT TGA TTG CA
	PPARG exon2	PPARG-ex2-F-1	TCT CCA GCA TTT CTA CTC CAC A
		PPARG-ex2-R-1	GTG GAT CCG ACA GTT AAG ATC AC
		PPARG-ex2-F-2	TCA AGC CCT TCA CTA CTG TTG
		PPARG-ex2-R-2	TGA AAC CGA CAG TAC TGA CAT TT
	PPARG exon3	PPARG-ex3-F-1	TGA ATG TCG TGT CTG TGG AGA
		PPARG-ex3-R-1	AGC TGG TCG ATA TCA CTG GAG
		PPARG-ex3-F-2	GTC GTG TCT GTG GAG ATA AAG C
		PPARG-ex3-R-2	GGA TTC AGC TGG TCG ATA TCA C
		PPARG-ex3-F-3	CCA ACT CCC TCA TGG CAA TT
		PPARG-ex3-R-3	CAA CAG CTT CTC CTT CTC GG
	PPARG exon4	PPARG-ex4-F	GTG ATC TTA ACT GTC GGA TCC AC
		PPARG-ex4-R	GGG GTG ATG TGT TTG AAC TTG A
	PPARG exon5	PPARG-ex5-F-1	ACA TAA AGT CCT TCC CGC TG
		PPARG-ex5-R-1	AGC AGG TTG TCT TGA ATG TCT
		PPARG-ex5-F-2	ACT CAT ACA TAA AGT CCT TCC CG
		PPARG-ex5-R-2	CTT GTA GCA GGT TGT CTT GAA TG
	PPARG EXON6	PPARG-ex6-F-1	TGC ACT GGA ATT AGA TGA CAG C
		PPARG-ex6-R-1	TGG AAG AAG GGA AAT GTT GGC
		PPARG-ex6-F-2	ACT GGA ATT AGA TGA CAG CGA C
		PPARG-ex6-R-2	CCC TCA GAA TAG TGC AAC TGG

Table S2. The antibodies for Western Blot.

Antibody	Dilution ratio	Company	Code No.
ACTB	1:5000	Proteintech	HRP-66009
Caspase-3 Antibody	1:1000	CST	9662
Caspase-8 (D35G2) Rabbit mAb	1:1000	CST	4790
Caspase-9 (C9) Mouse mAb	1:1000	CST	9508
